# Haze and inbound tourism: Empirical evidence from China

**DOI:** 10.3389/fpsyg.2022.1056673

**Published:** 2023-01-06

**Authors:** Wenzhi Wu, Xin Xia, Chunyu Cui, Fudong Qiu

**Affiliations:** ^1^Faculty of Economics and Management, East China Normal University, Shanghai, China; ^2^Glorious Sun School of Business and Management, Donghua University, Shanghai, China; ^3^Management College, Ocean University of China, Qingdao, China

**Keywords:** haze, inbound tourism, PM2.5, crowding-out effect, China

## Abstract

The impact of climate change on tourism has always been an important topic for research in the field of international tourism, and haze has been widely recognized as the primary negative factor affecting the development of inbound tourism in China. In this study, we first conduct a theoretical analysis of the mechanism through which haze influences the tourism industry, and then we empirically analyze the impact on China’s inbound tourism using surface particulate matter (PM2.5) concentrations as a proxy for haze, based on provincial panel data from 1998 to 2016. The empirical results show that haze not only has an inhibitory effect on inbound tourism, but also significantly reduces the average length of stay of international tourists. In addition, while there are significant regional differences in the crowding-out effect of haze pollution on inbound tourism, the effect varies depending on the origin of inbound tourists, exhibiting the greatest negative impact on inbound tourism from Taiwan and the smallest from foreign countries. Our research highlights that haze pollution can led to the change of human tourism behavior which enrich the literature on tourism and haze.

## Introduction

Since the period of reformation and opening up, China’s economy has grown steadily and rapidly, achieving a “growth miracle.” Subsequently, the “three highs” (high pollution, high energy consumption, and high emissions) incurred by the model of extensive economic development has led to the deterioration of the ecological environment. The more immediate environmental pollution caused by anthropogenic activities poses a serious threat to improving the quality of China’s economic growth. On September 27, 2016, the World Health Organization’s Global Air Pollution Report stated that China’s healthcare expenditure, labor time attrition, and additional welfare spending due to air pollution caused an annual loss of output equivalent to about 10% of the gross domestic product (GDP). Meanwhile, according to the China Tourism Academy (2016) issued by the China Tourism Research Institute, haze has become the main obstacle to the development of China’s inbound tourism market. In the same period, the *2016 China Tourism International Public Opinion Survey*, issued by China Tourism News and the Chinese Academy of Social Sciences, showed that in recent years the number of inbound tourists in China has continued to decline primarily because of the negative national image arising from air pollution. Specifically, haze severely limits the amount that visitors are prepared to travel within the country, effectively serving as the “killer” of China’s tourism development.

Haze is an aerosol system formed by a combination of a specific anomalous climate with numerous fine particles (dust, sulfuric acid, nitric acid, etc.) emitted by industrial production and human social activities. Fine particulate matter (PM2.5), which indicates an aerodynamic equivalent diameter of 2.5 microns or less, is considered the primary culprit for haze pollution. Despite the extent of the smoggy weather caused by haze pollution and the corresponding crisis in China’s image as a destination, academic explorations and theoretical constructions in this field are still inadequate. The literature mainly takes the systemic risk of haze to tourists’ activities in their destinations as its logical framework, or it uses the environmental determinism advocated by behaviorism as its theoretical basis. Nevertheless, previous studies the fail to address the key theoretical core, because as a new orientation of cognitive psychology, the “informed cognition” developed by tourists regarding destination haze does not follow the simple principle of “stimulus–response.” Instead, it follows the profound “mental construction” of environmental changes. In terms of economic effects, most developing countries have not established a sustainable smog monitoring system, and China itself only started monitoring domestic PM2.5 data in 2012. As a result, smog data are seriously inadequate. Therefore, empirical economic research about the crowding-out effect of haze on tourism is relatively scarce. Consequently, in addition to continuing to improve the theoretical basis with a better psychological framework, we provide empirical evidence to investigate the impact of haze on the development of tourism from an economic perspective, thereby expanding and deepening the understanding of the effect of climate change on tourism.

**Figure 1:**
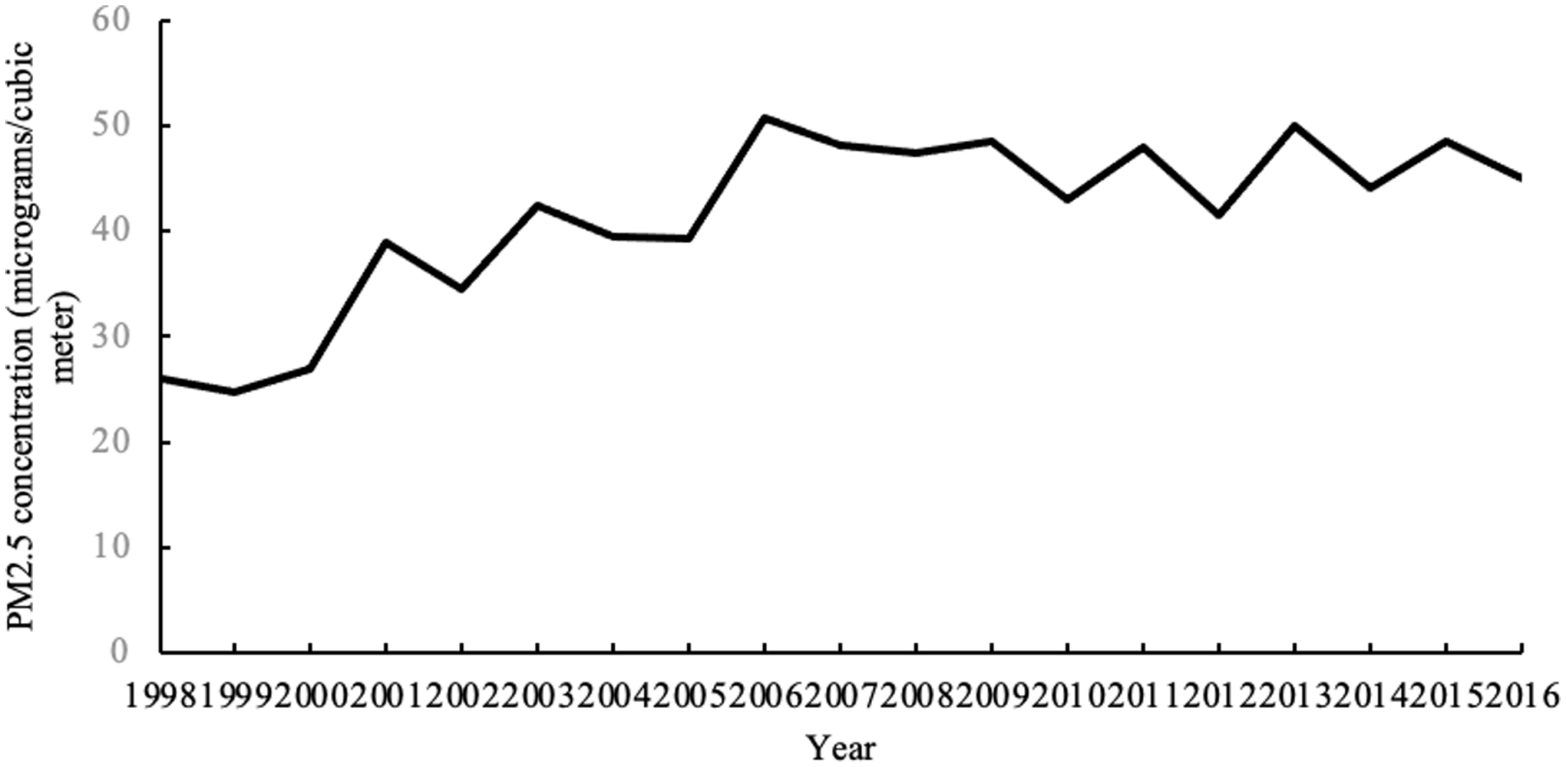
Annual average value of PM2.5 concentration in China from 1998 to 2016.

To address the absence of historical data on China’s PM2.5 readings and considering the availability of data and conventional academic practice, we as a source the data disclosed by the Socio-Economic Data and Applications Center (SEDAC) affiliated with the Center for International Earth Science Information Network (CIESIN) at Columbia University. The data are captured from mid-resolution satellite imaging that is carried out by the National Aeronautics and Space Administration (NASA). Based on the aerosol optical depth (AOD) measured by the Moderate-Resolution Imaging Spectroradiometer (MODIS) and the Multiangle Imaging Spectroradiometer (MISR), the data are converted into raster data showing the global average of PM2.5 surface concentrations (Donkelaar et al., 2010). We collect annual global mean PM2.5 concentrations from 1998 to 2016 (Figure 1). It should be noted that although the satellite monitoring data may be affected by meteorological factors, it can accurately reflect changes in the regional PM2.5 concentrations in the overall landscape. As the data belong to the surface source data, in contrast to the point source data of ground monitoring, they can be used as a credible measurement for haze pollution (Ma and Zhang, 2014; Shao et al., 2016; Huang, 2017).

In recent years, China’s inbound tourism market has experienced a downward trend. Besides political and economic factors, climate change has also invited more uncertainty that has, in turn, influenced the market. Meanwhile, academic research on the effect of haze on inbound tourism only started in recent years, and it lacks systematic analyses, especially in analyzing the economic influence of haze on tourism. Therefore, we test the relationship between inbound tourism development and haze pollution, as represented by the PM2.5 surface concentration data, empirically. Economic models are established based on panel data from 1985 to 2012 collected from 30 provincial-level units (excluding Hong Kong, Macao, Taiwan, and Tibet). Analyzing the data is expected to reveal the crowding-out effect of haze pollution, leading to a deeper understanding of the impact of haze on inbound tourism from an economic perspective.

In contrast with existing research, our main objective and contribution lies in the following three aspects.

First, we construct a theoretical framework based on environmental psychology to reveal the deep mechanism of why haze pollution can led to the change of human tourism behavior. It not only improve the core theoretical basis of the literature on haze and tourism but also deepen the academic understanding of tourists’ environmental perception in the field of tourism psychology. Second, we selected a more accurate indicators to measure the impact of haze on country’s inbound tourism, and decompose inbound tourists’ behaviors to more intuitively measure the crowding out effect of haze on inbound tourism. On the one hand, despite Xie et al. (2017) confirming the negative impact of haze on inbound tourism *via* the regression analysis of panel data from the perspective of exhaust, the selected indicators of atmospheric pollutants such as PM10, sulfur dioxide, and soot cannot adequately describe the effect of haze pollution, nor can they replace PM2.5 concentration as a more suitable indicator (Donkelaar et al., 2010). Accordingly, we combat this bias by choosing the PM2.5 concentration index to measure pollution more accurately. On the other hand, we added the variable of average stay time of tourists to decompose inbound tourists’ behaviors, thereby enriching the literature on tourism and haze. Third, we select a wider range of research object to explore the regional differences in the impact of haze pollution on inbound tourism. In recent years, Beijing has experienced a gradual decline in inbound tourism because of frequent smoggy weather. Academics therefore typically choose the city as a representative case in studying the relationship between smog and tourism. However, haze exhibits a characteristic of spatial agglomeration and diffusion due to the smaller particle size of PM2.5, which is uniformly distributed in the atmosphere, lingers for longer, and drifts farther. We expand the study sample to a provincial scale, which is consistent with the pervasiveness of smoggy weather in central and eastern regions. Panel data not only provide more dynamic behavior information about cross-section objects but also increase the accuracy of model estimation.

## Literature review

Despite the public concern over smoggy weather, academic contributions in this area are predominantly grey literature (i.e., produced by bodies that are not primarily concerned with academic publishing). As haze pollution is categorized as a phenomenon of air pollution, it is necessary to review and summarize the literature about the relationship between air pollution and tourism.

Climate condition is a notable attribute of tourist destinations, and climate-related tourism issues have received increased attention since the 1960s (Amelung et al., 2007). As concerns grew about global climate change in the 1980s, academic literature on the impact of climate change on tourism began to emerge (Scott et al., 2006). Against the backdrop of the growing scientific understanding of global climate change, in the 1990s the impact of climate change on tourism rapidly became a new field of knowledge in studies of international tourism (Hou et al., 2015). Generally, the literature separates the study of the impact of climate change on tourism into four categories:

1. The impact of climate change on tourism flows. Climate change affects the “push-pull” mechanism between tourists and destinations, thereby influencing the appropriateness of specific times and spaces for tourists’ travel (Scott et al., 2004).
2. The impact of climate change on tourists’ behaviors. Climate change is considered a sensitive factor that may affect tourists’ willingness to travel to certain destinations at certain times (Scott and Lemieux, 2010).
3. The impact of climate change on the tourist destination itself. Climate change has a negative impact on the ecology of the resources available, operating costs, image, and the types of activities available in tourist destinations.
4. The impact of climate change on the structure of the tourism industry. The environmental, social, and psychological changes caused by climate change are not conducive to the balance of supply and demand in the tourism industry (Pang et al., 2013).

The negative externalities of air pollution have now become a common research subject, resulting in a robust literary foundation. It is generally believed that air pollution has a strong negative impact on tourism and may even hamper regional economic development (Barker et al., 1982). From the perspective of the industry, tourists exposed to short- and long-term air pollution are more susceptible to acute health diseases (Neidell, 2004), and per Sönmez and Graefe (1998), the perception of risks to health negatively affects the travel motivations of potential visitors. Cheung and Law (2001) found that Asian tourists are more concerned about air quality in Hong Kong than are Western tourists. Zhang et al. (2015) analyzed the impact of haze perception on potential tourists in Beijing, suggesting that haze pollution not only affects tourists’ decision-making process, but it also causes potential tourists to cancel their travel plans. From the perspective of tourists, most studies have started to focus on testing and evaluating the effects of air pollution on destination tourism. Anaman and Looi (2000) used a Poisson regression to analyze air pollution phenomena through monthly tourist data for Brunei from January 1995 to September 1999. They found that haze pollution caused an approximately 28.7% monthly decline in tourist visits to Brunei and a direct loss of approximately 8 million Brunei dollars. Sajjad et al. (2014) conducted co-integration and Granger causality tests on time series data from 1975 to 2012 in South Asian, Middle Eastern, North African, Sub-Saharan African, and Asia-Pacific countries, and they found that air pollution was significant in most countries and had a negative effect on tourism. Air pollution has thus become a “nightmare” for tourism development. Deng et al. (2017) used spatial panel metrology technology to empirically analyze the panel data of 31 provinces in China from 2001 to 2013. They concluded that air pollution not only has a significant negative impact on the scale of inbound tourism, but it also exhibits a strong spatial spillover effect.

In recent years, especially with the pervasive nature of large-scale air pollution in mainland China, haze has become the most public, high-profile environmental issue. Concurrently, “haze research” has gained significant traction in the scientific community. Despite this, domestic research on the haze-tourism relationship is relatively scant. Not until 2015 did academic literature in this field begin to appear, with the main focus of such literature being on two areas:

1. The influence of haze on tourists. Gani and Clemes (2017) found that tourism is highly dependent on the weather conditions, it is more affected by haze weather than other industries. Although haze pollution is a long-standing problem in China, it was not recognized by the public until 2013 (van Donkelaar et al., 2015). Peng et al. (2016) conducted studies of Chinese mainland residents’, especially Beijing residents’, perceptions of haze and the related influences on tourists’ decision-making processes. Their results showed that mainland residents perceive strong health risks related to haze and that Beijing residents have a strong desire to escape haze. Haze pollution, therefore, both stimulates and strengthens the desire to travel for Beijing residents. Through surveys regarding attractions, such as the Great Wall, the Forbidden City, and the Summer Palace, Nan and Sun (2015) found that haze had a significant negative impact on factors influencing tourists’ decisions, including safety, health, willingness to travel, and choice of destination. Cheng et al. (2015), Li et al. (2015), and Zhang and Yan (2017) also confirmed these conclusions.
2. The impact of haze on the inbound tourism market. In recent years, studies pointed out that air environment is a main cause in inbound tourism; unsatisfactory air environment decreases the expectation and evaluation of inbound tourists, damages the international tourism image of the destination simultaneously (Awan et al., 2019; Zhang et al., 2019). In China, related works mainly paid attention to the specific destination (such as Beijing) with heavy haze pollution (Li et al., 2020), such as Yan and Zhang (2016a,b) found that hazy weather has a significant negative impact not only on Beijing but also on the national inbound tourism market; Gao and Ma (2016) and Xie et al. (2017) also used statistical experimentation to confirm the relevant conclusions. In addition, some researchers believed that the negative impact of haze weather on inbound tourism was temporary and tended to ease from a long-term perspective (Xu et al., 2020).

## Theoretical analysis

### Environment, psychology, and behavior

Air pollution poses a serious threat to human health, safety, and life expectancy. From the perspective of environmental psychology, air pollution not only jeopardizes human health but also negatively affects people’s inner psychology and external behavior (Calderón-Garcidueñas et al., 2015; Gauderman et al., 2015). Rotton et al. (1979) first revealed the adverse effects of air pollution and broke them down into direct and indirect effects. The direct effects refer to the damage to physical health and the related negative experiences. For example, epidemiological and toxicological laboratory studies have shown that air pollution damages the nervous system (Andersen et al., 2012), brain functionality (Brook et al., 2011), and cardiopulmonary functionality (Urman et al., 2014). In addition to physiological effects, air pollution’s psychological and behavioral effects are attracting greater attention due to their latent nature and slow manifestation of symptoms (Rotton and Frey, 1985). Air pollution not only impairs life satisfaction, but it can also reduce subjective well-being (Dolan and Laffan, 2016). In essence, air pollution leads to negative psychological effects in cognitive functioning (Ailshire and Clarke, 2014), emotions of depression and anxiety (Power et al., 2011), and the risk of self-abuse (Fleehart et al., 2014). The negative psychological effects caused by air pollution also make individual behavior and decision-making more conservative (Levy and Yagil, 2011; Xiang et al., 2017). First and foremost, the air pollution problem that smog represents is generally regarded as a risk factor for health that discourages people from engaging in daily outdoor activities and, in an effort to avoid environmental threats, even results in alterations to established travel plans. This in turn boosts negative emotions and lessens social productivity (Arvin and Lew, 2012).

### Haze negatively interferes with the decision-making process in tourism

Economic activities related to tourism depend on tourists moving between destinations. The process of receiving, sensing, and selecting information and then making decisions about destinations is a direct expression of tourism consumption behavior. In this process, one of the most critical behaviors is the act of selecting the destination before the tour. Tourism motivation (an individual factor) and the attributes of the destination (external inputs) are two prerequisites, two variables that constrain the tourism decision-making process (Heung et al., 2001; Galarza et al., 2002). It should be noted that we mainly reveal the mechanism through which haze influences the tourism decision-making process, working from the perspective of the effect of haze on the tourists and the destinations.

From the perspective of the tourists, motivation is not only the key factor in the decision-making process, but it is also a basic element affecting the tourism decision-making process. Behavioral economics provides an important theoretical basis for analyzing the impact of haze on tourism motivation. First, behavioral economics breaks with the assumption of “economic man” and “complete rationality” that is central to traditional economic theory, and it maintains that tourists exhibit “limited rationality.” Tourism motivation is not simply driven by interests but also by psychology and physiology, which have a compounded impact on motivation. Haze not only poses a potential risk to tourists’ health (Chen et al., 2014), but it also affects the emotions roused by tourists’ aesthetic psychology (Poudyal et al., 2013). These in turn hamper their internal travel motivations. Second, haze degrades the push-pull mechanisms formed by tourism psychology. Haze weakens the driving force behind tourism by reducing tourists’ willingness to travel. Furthermore, haze directly degrades the quality of the environment of tourist destinations, thereby weakening the attraction of those destinations to tourists (Kozak, 2002). In short, haze is a negative external stimulus that activates tourists’ intrinsic spiritual needs to generate motives for tourism (innovation and evasion), thus making tourism decision-making behavior selective (Um and Crompton, 1991). At the same time, because haze is a significant negative feature of tourism destinations, it further strengthens the choices of tourism decisions (Lohmann and Kaim, 1999). Third, in the classical model of the tourism decision-making process constructed by Woodside et al. (1989), the tourist’s knowledge of the destination is classified according to the emotional connection between the tourist, the destination, and the variables in the external context. Here, smog would be coded as an external context variable. The emotional connection between the tourist and the destination has a significant negative impact, thereby making the destination an “inert domain,” or even an “excluding domain,” in the tourist’s consciousness. This in turn changes the tourist’s preference for a specific destination and ultimately affects the motivation in the tourist’s “psychological account.”

Simultaneously, from the perspective of the destination, those with a positive image are more likely to be identified and selected by tourists when making decisions (Hunt, 1975). Tourist destinations are the ultimate target of tourism decision-making behavior. Tourists use limited information to form an image of tourism destinations, so the image of the destination is a key factor that influences tourism decision-making behavior (Pearce, 1982). Overall, the image of a tourist destination is the result of a series of beliefs, thoughts, and impressions that tourists form (Fakeye and Crompton, 1991). Haze worsens the quality of the environment of the destination, thus forming a negative image in tourists’ minds and in turn directly affecting their decision-making behavior.

At a deeper level, haze mainly exerts a negative influence on both the structure and formation of a destination’s image. In terms of structure, smog has a multi-dimensional effect on image. First, smog fosters tourists’ overall negative view of the psychological attributes of a destination, which destroys the “attribute-total chain” and “function-psychological chain” in composing the image of the destination (Echtner and Ritchie, 1991). Second, because it is a resource that is special to a destination, weather is an important component of the image of a tourist destination. Smog affects the emotional and overall formulation of the image of a destination by distorting tourists’ subjective view of its image. Eventually, it affects the behavioral intentions of tourists (Baloglu and McCleary, 1999). On the one hand, in terms of the formation of an image, Beerli and Martín (2004) described the mechanism as applied to tourist destinations as being inclusive of information sources (first-hand and second-hand) and individual factors (motivation, experience, and demographic variables). Through these means, smog not only causes tourists to have bad travel experiences and negative image perceptions, but it also inhibits and impairs their travel motivations and experiences. On the other hand, social representation theory shows that obtaining information through the media, practical experience, and social interaction serves as the basis for the construction of the image of a destination (Pearce 1996). Generally, because tourists’ knowledge of haze mainly comes *via* those three channels, smog subsequently has an adverse effect on the original image of a destination, especially after tourists have experienced smog there. Combined with the distorted subjective cognition mentioned above, a negative composite image of the destination is eventually formed (Tascim and Boylu, 2010).

### Haze damages the quality of travel experience

The travel experience is the essence of tourism research, and the “subjectivity” of tourists is the key to constructing a theory of tourism experience. Travel experience is collected through aesthetic activities and consumption in different places. Generally speaking, travel experience is the fundamental purpose of travel to a destination. Both phenomenology and Gestalt psychology emphasize that a holistic existence in the form of “field” is the key to the tourism experience for tourists (Xie and Peng, 2005). Accordingly, haze affects tourists’ decision-making process during the pre-tour stage, damages their travel experience throughout the trip, and even affects their post-tour behavior.

Smoggy weather makes the travel experience fragmented, in violation of Husserl’s phenomenological understanding of the holistic nature of the experience. Haze mainly undermines three aspects of tourists’ travel experience. First, in terms of the physical experience, the smoggy weather forces tourists to inhale particulate matter, potentially leading to respiratory and cardiovascular diseases (Chen et al., 2014). Smoggy weather causes the closure of traffic lanes, delays in transport, and road congestion, increasing travelers’ risks (Becken, 2012). Second, in terms of the psychological experience, smoggy weather decreases visibility at the destination, thus impairing the quality and mood of tourists’ travel experience. The scope and intensity of consumer activities and behavior (Mace et al., 2004), and the relevant potential risk factors caused by haze, can damage the intrinsic quality of tourism products and services, causing psychological stress, such as depression and anxiety. Finally, in terms of functional experience, the psychological pressure from the smoggy weather interferes with the aesthetic process as tourists gaze at the destination’s landscape and attractions. Psychological distance makes it difficult for tourists to enjoy experiences, such as through pleasure, nostalgia, and excitement (Urry, 1990). Semiotics shows that photography is an important manifestation of the visualization of the tourism experience. However, haze reduces visibility, and the acidic substances in it erode tourist attractions. These hurt the quality of photos, weaken appreciation of the landscape, and cripple tourists’ overall travel experience (Liu, 2004).

### The haze distorts tourist behavior

The mechanism by which haze affects tourists’ behaviors also follows the classical behavior analysis of “perception-attitude-behavior.” Haze causes tourists to attach higher costs to risks and uncertainties in the process of consuming a “destination” (Lundberg, 1995), resulting in the reduced value of tourist (customer) perceptions and satisfactions. This ultimately affects tourists’ travel intentions, as well as their willingness to revisit and recommend the destination (Ling et al., 2015). Taking the image of a tourist destination as an example, there is a positive feedback relationship between tourists’ subjective perception of the composite image of the destination and their loyalty to the destination. A negative image caused by haze will indirectly weaken tourists’ loyalty to a destination through the mediating effect of satisfaction.

For example, haze leads to the impairment of tourists’ experience of a destination, which causes dissatisfaction with it. Specifically, tourists’ negative perception of the destination’s image caused by haze directly affects their future travel decisions, thereby further reducing their willingness to revisit (Tascim and Boylu, 2010). And these negative effects can also cause spatial spillover effect, inbound tourists visiting the target province are influenced by the severity of haze weather in neighboring provinces (Tang et al., 2017). In addition, the image of the tourist destination has a special “core-edge” structure (Lai and Li, 2012), because the lexical distribution depicting the image of the tourist destination conforms to the long tail mode in the power law distribution (Pan and Li, 2011). Public perception of a destination’s image comes from a few high-frequency words, and as such, “haze” crystalizes a destination’s negative image and tourists’ negative word-of-mouth，especially given today’s fast-paced mass media. In the above process, fewer intentions to revisit and negative word-of-mouth combine to make it difficult for destinations to shape a positive image (Mao and Zhang, 2014), ultimately reducing the number of potential tourists.

## Methodology

### Model construction

According to the literature, China’s inbound tourism market is foundationally constrained by three factors: tourism endowment, external conditions, and environmental conditions (Berenyi, 1980; Turner and Witt, 2001). As a special environmental condition, haze is chosen as the core explanatory variable of this study. Therefore, to test and evaluate the effect of haze on the development of the inbound tourism market, the following benchmarking model is constructed:


(1) $$InTOUR_{it}=\beta_{0}+\beta_{1}\ln PM_{it}+\Theta X_{it}^{a}+\Psi X_{it}^{b}+\Omega X_{it}^{c}+\mu_{i}+\varepsilon_{it}$$

where *i* indicates the province, *t* indicates the time, *μ_i_* is the non-observed individual factor that does not change with time, *ε_it_* is a random disturbance term, *PM* indicates smog pollution, and *TOUR* is the scale of inbound tourism. The objective of this study is to analyze the impact of haze on the development of the inbound tourism market. As the theory of tourism economics suggests, the development of that market is a function of three factors: tourism resource endowment, economic and social development, and institutional environmental quality. Therefore, in addition to the core explanatory variables of the measurement model, other explanatory variables must also be controlled to avoid the endogenous bias caused by the omission of variables and random disturbances. The classical empirical literature on the factors affecting tourism (Prideaux, 2005; Turaev, 2010) is used to classify the model control variables into three categories: $X_{it}^{a}$ represents the set of intrinsic endowment control variables in tourism; $X_{it}^{b}$ represents the set of economic and social development control variables; and $X_{it}^{c}$ represents the set of institutional environmental quality control variables. To reduce heteroscedasticity, all control variables take the form of a natural logarithm.

### Variable construction

The scale of inbound tourism is the explained variable, which is measured by the number of inbound tourists (in millions, *TTP*) and the average length of stay (in days, *ATD*) of inbound tourism. Haze is the core explanatory variable. Although China added PM2.5 as a monitoring indicator in the Environmental Air Quality Standard (New Revision) as of February 2012, the indicator only covers a limited number of cities. Therefore, we use data based on the global PM2.5 surface concentrations observed by remote sensing technology at Columbia University’s Center for Social and Economic Data and Applications. We use ArcGIS software combined with China’s provincial administrative region vector map to analyze the global PM2.5 surface concentration remote sensing raster data of 30 provinces in China from 1998 to 2012 (excluding Hong Kong, Macao, Taiwan, and Tibet).

In terms of control variables, the intrinsic endowments of tourism include:

1. Tourism resource endowment. As a primary productive factor of tourism products, tourism resources effectively characterize the intensity of attraction and represent the core conditions for tourism development. To reflect tourism resource endowment, we comprehensively weight the following factors: the number of World Heritage sites owned by the province (weight 4); the number of excellent tourist cities (weight 3); the number of national scenic spots (weight 2); and the number of 4A and 5A tourist attractions (weight 1; Wang et al., 2016).
2. Tourism enterprises’ physical capital and employment. Fixed assets and labor are the basic inputs for the development of tourism enterprises, and these directly determine the scale of the output of tourism enterprises. This output is measured by the value of the fixed assets of the tourism enterprises (100 million yuan) and the number of employees in those enterprises (100 persons).
3. Tourist reception scale. The scale of tourism reception reflects the tourist capacity of the source of visitors to the destination, which is a number that reflects the capacity that is needed to maintain the economic operation of tourism at the destination. It is measured by the sum of the number of star hotels and travel agencies owned by the province.

The economic and social development categories include:

1. Economic growth: The higher the level of economic growth, the greater the intensity of the destination’s investment in tourism infrastructure. We measure the GDP *per capita* after the deflator.
2. Urbanization: The urbanization process can optimize the spatial structure of tourism destinations, which is conducive to the spreading out of tourism flows. The urban population is measured in proportion to the total population.
3. Industrial structure: The resource allocation effect of changes to the industrial structure will promote the transfer, flow, and agglomeration of production factors, such as cross-industry capital, labor, and technology to the tourism sector, thus providing an important source for tourism development. The value added of tertiary industry is measured in proportion to GDP.

The institutional environmental quality categories include:

1. Foreign economic engagement: The higher the level of foreign direct investment, the stronger the destination’s ability to outsource. A positive international image has also become an important factor in attracting cross-regional tourism. It is measured by how much foreign direct investment is used as a proportion of GDP.
2. Marketization: The marketization process can show the extent of freedom enjoyed by the market economy in the destination market, reflecting how good the consumer environment is for tourists. The provincial marketization index calculated by Fan et al. (2011) and Wang et al. (2017) is used for measurement.

### Data sources

This study is based on 30 provinces in China, excluding Hong Kong, Macao, Taiwan, and Tibet. To maintain data availability and continuity, the study integrates Columbia University’s Battle Institute data of global PM2.5 readings during a sample period from 1998 to 2016. Other tourism-related data come from the The Yearbook of China Tourism Statistics (1999–2017); other raw data are derived from the China Statistical Yearbook (1999–2017) and the China Industrial Economics Statistical Yearbook (1999–2017).

## Empirical results

### Baseline regression

#### The full sample estimation

To detect multicollinearity before the regression, the variance inflation factor (VIF) is used to test whether the multicollinearity problem is solved. The test result show the VIF statistics is 2.47, which means that the variance of the coefficient has not been inflated by multicollinearity, and there is no multicollinearity problem between the control variable and interesting variables. Meanwhile, this study utilized clustering robust standard error to account for heteroscedasticity of unexplained changes in the model. It is a special kind of robust standard error that can explain heteroscedasticity of “clustered” observations such as countries, industries, or individuals. Because of the correlation between the random disturbance terms of the same individual in different years, the clustering robust standard error can better capture the characteristics of the intra-group correlation, so as to obtain a consistent estimate of the true standard error. The impact of haze on the development of the inbound tourism market in the full-sample model results displayed in Table 1. Taking the number of inbound tourists as the explained variable, as shown in Table 1, column (1) lists the ordinary least squares (OLS) regression results without controlling for such variables as provinces, year fixed effects, and economic and social development, as well as institutional environmental quality. The haze regression coefficient is negative and significant at the 1% level, which is consistent with theoretical expectations and indicates that the gradual increase of haze pollution in recent years has to a certain degree inhibited the growth of China’s inbound tourism.

**Table 1:** Full sample estimation results.

Variables	lnTTP				lnATD			
	(1)	(2)	(3)	(4)	(5)	(6)	(7)	(8)
lnPM	−0.602^***^ (0.062)	−0.389^***^ (0.013)	−0.556^***^ (0.087)	−0.602^***^ (0.024)	−0.123^**^ (0.031)	−0.115^**^ (0.014)	−0.119^***^ (0.025)	−0.128^***^ (0.016)
lnTRS		0.223^***^ (0.057)	0.276^***^ (0.053)	0.166^***^ (0.032)		0.101^***^ (0.019)	0.097^***^ (0.022)	0.044^***^ (0.012)
lnTCK		0.011 (0.034)	0.002 (0.045)	0.003 (0.011)		−0.013 (0.012)	0.002 (0.013)	0.001 (0.015)
lnTRB		−0.058^*^ (0.023)	−0.032 (0.023)	−0.026 (0.033)		−0.023 (0.015)	−0.018 (0.011)	−0.024 (0.018)
lnTHP		0.043^**^ (0.101)	0.253^**^ (0.12)	0.301^**^ (0.031)		0.033^**^ (0.011)	0.002 (0.013)	0.002 (0.021)
lnGDP			1.196^***^ (0.112)	1.055^***^ (0.27)			0.401^***^ (0.106)	0.426^***^ (0.017)
lnURB			−0.111 (0.023)	−0.015 (0043)			0.187^***^ (0.021)	0.188^***^ (0.019)
lnSTR			0.612^***^ (0.025)	0.451^**^ (0.018)			0.426^***^ (0.016)	0.508^***^ (0.102)
lnFDI				0.075^***^ (0.012)				0.013^***^ (0.032)
lnMKT				0.603^***^ (0.022)				0.127^*^ (0.005)
Province fixed effects	No	Yes	Yes	Yes	No	Yes	Yes	Yes
Year fixed effects	No	Yes	Yes	Yes	No	Yes	Yes	Yes
Obs.	450	450	450	450	450	450	450	450
*R* ^2^	0.625	0.713	0.726	0.751	0.287	0.242	0.22	0.564

*, **, and *** indicate 10, 5, and 1% significance levels, respectively; standard deviations are in parentheses.

In light of provincial differences and considering the influence of trends over time, related variables and fixed effects are controlled for starting from the estimates in column (2). The regression results in columns (2) to (4) show that the haze regression coefficient is significant and maintains a value between −0.4 and − 0.6, indicating that haze pollution has caused some degree of change by inhibiting inbound tourists. The empirical results are consistent with the findings of Xu et al. (2020). In contrast to previous studies, which have only used sulfur dioxides, nitrogen oxides, or smoke (powder) dusts as the proxy variables of haze pollution, we provide a more accurate and generalized estimation by using PM2.5 concentrations to reflect the degree of haze pollution. By selecting the average length of stay (in days) of inbound tourism as the explained variable, the regression results confirm that haze pollution reduces inbound tourists’ average length of stay. In general, haze pollution has a crowding-out effect on the development of China’s inbound tourism market, which in turn significantly reduces the scale of China’s inbound tourism market.

For control variables, the greater the endowment of tourism resources, the stronger the attraction exerted on inbound tourism. The regression coefficient of fixed assets of tourism enterprises is virtually non-significant. The inbound tourism market is mainly comprised of activities such as sight-seeing, ecotourism, and cultural tourism, activities that are ultimately of higher quality but do not necessarily require heavy fixed assets. In addition, the quality of investment in the fixed assets of tourism enterprises may not meet the needs of the inbound tourism market. The number of people employed in tourism is also non-significant. This is due to the fact that the quality of the statistics on the number of employees in China’s tourism enterprises has changed many times, making this indicator chronically inconsistent. The regression coefficient of the tourism reception scale is significant, indicating that improvement in tourism reception is a contributing factor to the development of the inbound tourism market. The regression coefficient of economic growth is highly significant, indicating that general economic growth plays a significant role in supporting the inbound tourism market. The impact of urbanization on the inbound tourism market is open to question. Although urbanization can provide a sufficient investment base for the inbound tourism market, excessive or extensive urbanization homogenizes the quality of what is experienced by inbound tourists. The industrial transformation and upgrade of the modern service industry creates enough room for growth in the inbound tourism market. The more open the external market, the greater the quality of the system and the more inbound tourists tend to travel to those destinations.

#### Regional estimation results

Due to regional disparities in meteorological conditions, energy structure, and the industrial system, the intensity of haze pollution in China’s provinces varies. Therefore, to investigate the differences in the effects of smog pollution on the inbound tourism market in different areas, we first estimate samples for the south and the north, and then the eastern, central, and western samples. The results are listed in Table 2. Whether the explained variables are the number of inbound tourists or the average length of stay, the regression coefficients of haze and smog in the south and north are significantly negative, whereas the comparison of absolute values shows that haze pollution in the north affects the development of the inbound tourism market more than it does in the south. From this we can conclude that the crowding-out-effect is greater in the north than in the south. A probable reason for this is that relatively low energy efficiency in the north contributes to the “three highs” (high input, high energy consumption, and high pollution) in that area and that winter heating in the entire region also exacerbates the problem. PM2.5 differs from PM10 and other smog “culprits” in that it consists of small particles, is long lasting, and has wide distribution, especially the more toxic substances, and therefore poses a serious threat to physical and mental health, as well as to the experience of inbound tourists.

**Table 2:** Regional estimation results.

Variables	lnTTP					lnATD				
	North	South	East	Central	West	North	South	East	Central	West
lnPM	−0.634^**^ (0.120)	−0.577^***^ (0.171)	−0.309^**^ (0.382)	−0.147^**^ (0.405)	0.469 (0.192)	−0.166^**^ (0.066)	−0.067^*^ (0.133)	−0.231^**^ (0.179)	−0.184^*^ (0.223)	−0.221^***^ (0.054)
lnTRS	0.154 (0.122)	0.099 (0.092)	0.183^*^ (0.089)	0.172 (0.176)	0.381^**^ (0.161)	0.177^***^ (0.067)	0.017 (0.043)	0.039 (0.092)	0.109 (0.097)	0.043 (0.059)
lnTCK	0.117 (0.069)	0.005 (0.047)	0.171 (0.079)	0.037 (0.074)	0.083 (0.086)	0.026 (0.037)	0.039^*^ (0.022)	0.004 (0.068)	0.004 (0.051)	−0.011 (0.027)
lnTRB	−0.069 (0.068)	−0.012 (0.054)	−0.105 (0.082)	−0.032 (0.076)	−0.224 (0.132)	−0.019 (0.037)	−0.079 (0.025)	−0.059 (0.057)	−0.096^*^ (0.048)	−0.037 (0.034)
lnTHP	0.055^**^ (0.121)	0.232^**^ (0.108)	0.120 (0.259)	0.373^*^ (0.189)	0.494^**^ (0.230)	0.025^**^ (0.066)	0.065^**^ (0.051)	0.023^*^ (0.127)	0.095 (0.205)	0.054 (0.049)
lnGDP	1.151^***^ (0.454)	0.740^***^ (0.244)	0.594^**^ (0.284)	0.172^*^ (0.737)	1.938^***^ (0.628)	0.969^***^ (0.248)	0.316^***^ (0.114)	0.521^*^ (0.285)	0.268^*^ (0.453)	0.071^*^ (0.197)
lnURB	−0.125 (0.236)	−0.087 (0.106)	0.039 (0.184)	0.410 (0.289)	−0.197 (0.317)	0.482^***^ (0.129)	0.174^***^ (0.049)	0.260^**^ (0.128)	0.104 (0.127)	0.003 (0.083)
lnSTR	0.314^**^ (0.470)	0.153^***^ (0.302)	0.989^**^ (0.403)	0.745 (0.522)	0.194 (0.438)	0.574^**^ (0.257)	0.236^*^ (0.142)	0.099 (0.398)	0.604^*^ (0.312)	0.782^***^ (0.166)
lnFDI	0.067 (0.050)	0.075 (0.047)	0.114^*^ (0.062)	0.052 (0.070)	0.123^**^ (0.053)	−0.001 (0.027)	−0.009 (0.022)	0.028 (0065)	0.008 (0.033)	0.030 (0.021)
lnMKT	0.552^***^ (0.201)	1.111^***^ (0.214)	0.242 (0.294)	1.403^***^ (0.374)	0.642^*^ (0.327)	0.155^***^ (0.110)	0.126^**^ (0.100)	0.041^*^ (0.220)	0.127 (0.308)	0.008 (0.094)
观测值	240	210	165	105	165	240	210	165	105	165

*, **, and *** indicate 10, 5, and 1% significance levels, respectively; standard deviations are in parentheses.

The regression coefficients of haze in the eastern and central provinces are also significantly negative, whereas haze pollution in the eastern provinces has a greater crowding-out effect than in the central region. In the western provinces, the negative effects on the average lengths of stay of inbound tourists are significant, but the impact on tourist arrivals is not significant. The conclusions of this portion of the study are consistent with those of Xie et al. (2017). A primary reason for this distinction is that concentrations of PM2.5 in the eastern provinces is higher than in the central and western provinces (Cai et al., 2017). This higher intensity of haze pollution has a greater risk of blocking the development of the inbound tourism market in the eastern provinces. In addition, both the size and the usage of the inbound tourism market in the east are higher than in the central and western provinces, indicating that inbound tourists have higher expectations and feel the effects of pollution more intensely. Inbound tourism in the western provinces is relatively small, but the attractiveness of tourism resources in the western provinces is unique. Although the relatively low level of smog pollution does not significantly affect the number of inbound tourists, it still reduces the average lengths of stay of inbound tourists.

### Robustness test

By way of introduction, the baseline regression results in this study support the theoretical analysis. To test the robustness of the regression results, we replace the core explanatory variables with sulfur dioxide and smoke (powder) dust as proxies for haze pollution. To solve the potential endogeneity problem in this model, we additionally investigate the exogenous variable as the instrumental variable related to the endogenous explanatory variable but not related to the error term. This instrumental variable is then regressed on the model. To this end, we refer to the practice of the mainstream literature, selecting the lagged value of haze pollution as the instrumental variable and estimating it using the fixed effect two-stage least squares method (FE-2SLS) and the fixed effect generalized moment estimation method (FE-FMM), respectively. The regression results are shown in Tables 3, 4, respectively. Columns (1) and (3) of Table 3 show that the regression coefficient of sulfur dioxide is significantly negative, and columns (2) and (4) also report a significantly negative coefficient of smoke (powder) dust. These results both indicate that emissions of sulfur dioxide and smoke (powder) dust also have a negative effect on the development of China’s inbound tourism market. Table 4 reports the regression results of the two-panel instrumental variables, showing that the haze regression coefficient remains negative at different significance levels and indicating that after controlling for possible endogeneity, the crowding-out effect of haze pollution on the development of the inbound tourism market still exists. At the same time, the Kleibergen-Paaprk LM statistic and the Kleibergen-Paaprk Wald F statistic both significantly reject the null hypothesis of “insufficient instrumental variable recognition” and “weak instrumental variables” at the 1% level, and the Hansen J statistic is not significant, indicating the exogenous validity of the instrumental variables selected by the model.

**Table 3:** Robustness tests.

Variables	lnTTP		lnATD	
	(1)	(2)	(3)	(4)
$\ln SO_{2}$	−0.058^**^ (0.080)		−0.121^**^ (0.041)	
lnSD		−0.205^***^ (0.063)		−0.081^**^ (0.033)
Control variables	Yes	Yes	Yes	Yes
Province effect	Yes	Yes	Yes	Yes
Year effect	Yes	Yes	Yes	Yes
Obs.	450	450	450	450
*R* ^2^	0.669	0.677	0.434	0.454

*, **, and *** indicate 10, 5, and 1% significance levels, respectively; standard deviations are in parentheses.

**Table 4:** Instrumental variables regression.

Variables	lnTTP		lnATD	
	(1) FE-2SLS	(2) FE-GMM	(3) FE-2SLS	(4) FE-GMM
lnPM	−0.668^***^ (0.201)	−0.790^***^ (0.197)	−0.188^*^ (0.111)	−0.128^*^ (0.108)
Control variables	Yes	Yes	Yes	Yes
Province fixed effects	Yes	Yes	Yes	Yes
Year fixed effects	Yes	Yes	Yes	Yes
Kleibergen-Paaprk LM	50.112^***^	50.112^***^	50.112^***^	50.112^***^
Cragg-Donald Wald F	215.156^***^	215.156^***^	215.156^***^	215.156^***^
Kleibergen-Paaprk Wald F	494.516^***^	494.516^***^	494.516^***^	494.516^***^
Hansen J	0.531	0.531	0.454	0.454
Obs.	390	390	390	390
*R* ^2^	0.676	0.671	0.179	0.173

* and *** indicate 10 and 1% significance levels, respectively; standard deviations are in parentheses.

### The regression-based decomposition of the tourism market structure

To reveal the difference in the tourism market structure and to understand the sensitivity to haze pollution of the major inbound source markets, we categorize tourists from the inbound source markets as foreigners, Hong Kong, Macau, and Taiwanese. Columns (1) to (4) in Table 5 show that the smog regression coefficient is significantly negative, and the absolute value of the coefficient increases from Columns (1) to (4). This indicates that haze pollution negatively affects foreigners, Hong Kong, Macao, and Taiwanese inbound tourists and that the inhibition effect increases from foreigners to Hong Kong, Macao, and Taiwanese tourists. Columns (5) to (8) of Table 5 also show the effect on the average lengths of stay for foreigners, Hong Kong, Macao, and Taiwanese inbound tourists. With the absolute value of the coefficient rising from Columns (5) to (8), it can be concluded that haze pollution has the greatest negative impact on Taiwan’s inbound tourism market, and it has the least impact on the foreign inbound tourism market.

**Table 5:** Regression results based on the tourism market structure.

Variables	lnTTP				lnATD			
	(1) Foreigners	(2) Hong Kong	(3) Macau	(4) Taiwan	(5) Foreigners	(6) Hong Kong	(7) Macau	(8) Taiwan
lnPM	−0.205^***^ (0.066)	−0.223^**^ (0.092)	−0.253^***^ (0.071)	−0.297^***^ (0.008)	−0.151^***^ (0.049)	−0.188^***^ (0.056)	−0.253^***^ (0.071)	−0.312^***^ (0.059)
Control variables	Yes	Yes	Yes	Yes	Yes	Yes	Yes	Yes
Province fixed effects	Yes	Yes	Yes	Yes	Yes	Yes	Yes	Yes
Year fixed effects	Yes	Yes	Yes	Yes	Yes	Yes	Yes	Yes
Obs.	450	450	450	450	450	450	450	450
*R* ^2^	0.701	0.532	0.491	0.614	0.528	0.455	0.491	0.581

** and *** indicate 5 and 1% significance levels, respectively; standard deviations are in parentheses.

## Conclusion and policy implications

### Conclusion

With the continuous growth of the global tourism market, international tourism service trade has become ever more frequent. As a basic form of service export, inbound tourism can accumulate national wealth. And inbound tourism has always been the main focus of destination management. Classical tourism theory insists that the environment of a destination is an important limiting factor in the development of the inbound tourism market. In recent years, China has experienced unprecedented haze, which not only poses a serious threat to people’s well-being but also hinders economic and social development. During this same period, the inbound tourism market has also shown a downward trend, with haze being named as the main culprit. Despite this, few academic studies have delved deep into the issue, with a notable deficit in economic analysis. In light of this, we first analyze the environmental psychology behind the influence of smog on tourism and then carefully identify the effect of haze pollution over time through surface measurements of concentrations of PM2.5. Based on provincial panel data from 1998 to 2016, an empirical test is conducted. The results suggest that haze pollution not only inhibits the growth of inbound tourism on the mainland but also reduces the average lengths of stay of inbound tourists, which is a further expansion of researches of Xie et al. (2017) and Li et al. (2020). In general, haze pollution significantly reduces the scale of inbound tourism, a conclusion verified through a robustness test. Moreover, regional differences, as well as disparities between different customer structures, are observed in the crowding-out effect of haze on the market. The negative impact of smog pollution in the northern and eastern markets is found to be greater in the south, central, and western regions and the negative impact of smog pollution on inbound tourists from Taiwan is stronger than on Macao, Hong Kong, and foreign inbound tourists, avoided the limitation of analyzing only specific cities in China.

### Policy implication

The policy implications of this study are intuitive and profound. To alleviate the negative impact of smog pollution on tourism, two approaches make sense. One concerns the control of haze pollution and the other concerns improving the quality of inbound tourism. To control smog, it is imperative to formulate and implement an effective, scientific reduction policy. For example, long-term environmental administrative regulation could be strengthened to force industrial structures to upgrade and require the optimization of energy structures. An assessment of the quality of the environment could also be considered when appraising city officials for promotion. We can also actively encourage innovation in green technology through financial and tax incentives. It is noteworthy that a change in tactics in economic development is fundamental to curbing haze pollution.

Continual improvement in the quality of inbound tourism programs can be carried out in the following three ways. First, we can establish an indirect service system for inbound tourism. Besides meeting tourists’ basic needs with direct tourism services, such as sight-seeing, ecotourism, and cultural tourism, it is also necessary to provide and improve content, such as business exhibitions, academic exchanges, cultural communications, and scientific research activities. Second, we can expand revenue sources for the inbound tourism service system. We must explore and protect traditional service demands made by inbound tourists, including postal, telecommunications, foreign exchange, medical insurance, education, and training services at their destinations. Finally, we suggest establishing a knowledge-intensive inbound tourism service system. In contrast to traditional labor-intensive trade, capital-intensive service trade deepens the integration of information technology with tourism and focuses on the development of international smart tourism. This would help enhance the quality of inbound tourists’ experience. In essence, the continued development of China’s market in inbound tourism can be ensured by reducing haze emissions and improving the quality of inbound tourism services.

## Data availability statement

The original contributions presented in the study are included in the article/supplementary material, further inquiries can be directed to the corresponding author.

## Author contributions

WW, CC, and XX were responsible for writing the preliminary research review and data analysis. FQ was responsible for data collection and writing policy recommendations. All authors contributed to the article and approved the submitted version.

## Conflict of interest

The authors declare that the research was conducted in the absence of any commercial or financial relationships that could be construed as a potential conflict of interest.

## Publisher’s note

All claims expressed in this article are solely those of the authors and do not necessarily represent those of their affiliated organizations, or those of the publisher, the editors and the reviewers. Any product that may be evaluated in this article, or claim that may be made by its manufacturer, is not guaranteed or endorsed by the publisher.
